# Risk Prediction of Major Adverse Cardiovascular Events Within One Year After Percutaneous Coronary Intervention in Patients With Acute Coronary Syndrome: Machine Learning–Based Time-to-Event Analysis

**DOI:** 10.2196/81778

**Published:** 2025-11-27

**Authors:** Hong-Jae Choi, Changhee Lee, Hack-Lyoung Kim, Youn-Jung Son

**Affiliations:** 1 Department of Thoracic and Cardiovascular Surgery, Seoul Metropolitan Government-Seoul National University Boramae Medical Center, Seoul National University College of Medicine Seoul Republic of Korea; 2 Department of Artificial Intelligence, Korea University Seoul Republic of Korea; 3 Division of Cardiology, Department of Internal Medicine, Seoul Metropolitan Government-Seoul National University Boramae Medical Center, Seoul National University College of Medicine Seoul Republic of Korea; 4 Red Cross College of Nursing, Chung-Ang University Seoul Republic of Korea

**Keywords:** acute coronary syndrome, machine learning, major adverse cardiovascular events, percutaneous coronary intervention, predictive models, survival analysis

## Abstract

**Background:**

Patients with acute coronary syndrome (ACS) who undergo percutaneous coronary intervention (PCI) remain at high risk for major adverse cardiovascular events (MACE). Conventional risk scores may not capture dynamic or nonlinear changes in postdischarge MACE risk, whereas machine learning (ML) approaches can improve predictive performance. However, few ML models have incorporated time-to-event analysis to reflect changes in MACE risk over time.

**Objective:**

This study aimed to develop a time-to-event ML model for predicting MACE after PCI in patients with ACS and to identify the risk factors with time-varying contributions.

**Methods:**

We analyzed electronic health records of 3159 patients with ACS who underwent PCI at a tertiary hospital in South Korea between 2008 and 2020. Six time-to-event ML models were developed using 54 variables. Model performance was evaluated using the time-dependent concordance index and Brier score. Variable importance was assessed using permutation importance and visualized with partial dependence plots to identify variables contributing to MACE risk over time.

**Results:**

During a median follow-up of 3.8 years, 626 (19.8%) patients experienced MACE. The best-performing model achieved a time-dependent concordance index of 0.743 at day 30 and 0.616 at 1 year. Time-dependent Brier scores increased and remained stable across all ML models. Key predictors included contrast volume, age, medication adherence, coronary artery disease severity, and glomerular filtration rate. Contrast volume ≥300 mL, age ≥60 years, and medication adherence score ≥30 were associated with early postdischarge risk, whereas coronary artery disease severity and glomerular filtration rate became more influential beyond 60 days.

**Conclusions:**

The proposed time-to-event ML model effectively captured dynamic risk patterns after PCI and identified key predictors with time-varying effects. These findings may support individualized postdischarge management and early intervention strategies to prevent MACE in high-risk patients.

## Introduction

Acute coronary syndrome (ACS) is a critical and sudden presentation of underlying coronary artery disease (CAD); it requires timely intervention to reduce the risk of adverse outcomes and mortality [1-5]. The primary method of revascularization used in the management of ACS is percutaneous coronary intervention (PCI) because it promptly improves coronary blood flow and minimizes myocardial damage [4,6]. Although timely PCI enhances acute survival, patients often face challenges such as poor medication adherence, insufficient lifestyle modification, and residual psychosocial stress following discharge [7,8]. They also remain at substantial risk for major adverse cardiovascular events (MACE), particularly within the first year after PCI [9-12]. MACE are typically defined as the composite outcome of cardiovascular death, nonfatal myocardial infarction, stroke, or unplanned revascularization [4,13]. Approximately 14.8% to 34.4% of patients who have ACS and undergo PCI experience MACE within the first year [14,15]. The MACE risk arises from the combined influence of multiple contributing factors, including demographic characteristics, lifestyle-related factors, comorbidities, and medication-related factors such as polypharmacy and medication use patterns [2,11].

Several risk stratification tools have been developed to estimate the MACE risk in patients with ACS; among them, the Thrombolysis in Myocardial Infarction Risk Score and the Global Registry of Acute Coronary Events Risk Score are the most widely used [16]. Although these tools provide valuable support for initial triage and decision-making, they have several critical limitations. First, they are based on a limited set of clinical variables; as such, they fail to capture the multifactorial nature of the MACE risk [7,16]. Second, they do not reflect temporal variations in MACE risk after discharge; instead, they rely on static, single timepoint assessments [1,16]. Additionally, these traditional models are built on simplified linear statistical assumptions, which may inadequately reflect the complex, nonlinear relationships between risk factors [1,16]. Moreover, when risk stratification scores are used, manual data entry is required, thereby reducing practicality and limiting routine use in real-world clinical settings; it also lacks adaptability to incorporate emerging clinical guidelines [17,18].

Some of these limitations have been addressed by recent advances in machine learning (ML). For instance, ML models have improved the ability to capture complex nonlinear relationships, automate risk prediction without manual data entry, and facilitate faster updates consistent with evolving clinical knowledge [19,20]. Despite these improvements, most ML models developed for predicting MACE in patients with ACS who undergo PCI continue to rely on a limited set of clinical variables and fail to incorporate time-to-event information [21-23]. As a result, they overlook the dynamic nature of MACE risk over time. Particularly, most existing ML studies have framed MACE prediction as a binary classification problem [21-23] and focused solely on whether an event will occur within a fixed time frame. With this approach, right-censored patients are often excluded, thereby introducing a potential risk of bias in the prediction model because of the underlying covariate shift [24]. Moreover, such models have a limited ability to estimate the probability of event occurrence across clinically relevant time intervals [25]. Consequently, their applications are limited in guiding temporally informed clinical interventions.

Time-to-event ML models that combine the strengths of survival analysis and ML offer a compelling solution to overcome the limitations of traditional methods. They can be used to estimate event occurrence probabilities over time without requiring the proportional hazards assumption of traditional Cox models; consequently, they provide greater flexibility for modeling complex real-world scenarios [24,25]. Accordingly, we aimed to develop a time-to-event ML model for predicting MACE in patients who had ACS and underwent PCI. Our model incorporated clinical, demographic, and medication-related variables and used time-to-event information to enable dynamic risk estimation. Furthermore, we aimed to identify how key risk factors contributed to MACE at different time intervals by evaluating the time-varying importance of predictive variables, thereby supporting personalized and time-sensitive clinical decision-making.

## Methods

### Study Design

This retrospective cohort study followed the Transparent Reporting of a Multivariable Prediction Model for Individual Prognosis or Diagnosis + Artificial Intelligence (TRIPOD+AI) checklist to uphold methodological rigor and ensure transparent reporting of the ML–based predictive model [26]. In addition, we adhered to the Strengthening the Reporting of Observational Studies in Epidemiology (STROBE) checklist [27]. Details of the TRIPOD+AI checklist are provided in Multimedia Appendix 1, and the STROBE reporting items are presented in Multimedia Appendix 2.

### Data Sources and Patients

This study was conducted using electronic medical record (EMR) data from a single tertiary hospital in Seoul, South Korea. Patients diagnosed with ACS and subjected to PCI between January 1, 2008, and December 31, 2020, were identified. The EMR data included demographic, lifestyle, clinical, and medication-related information. The extracted data were fully anonymized to protect patient privacy. PCI procedures were performed in accordance with current guideline recommendations [4,6,27].

The patients were eligible for inclusion if they met the following criteria (1) aged 19 years or older and (2) underwent PCI for the first time. The exclusion criteria were as follows (1) patients who experienced MACE during the index hospitalization, (2) patients without follow-up records after discharge, and (3) patients with missing or erroneous outcome records for MACE after discharge.

**Figure 1:**
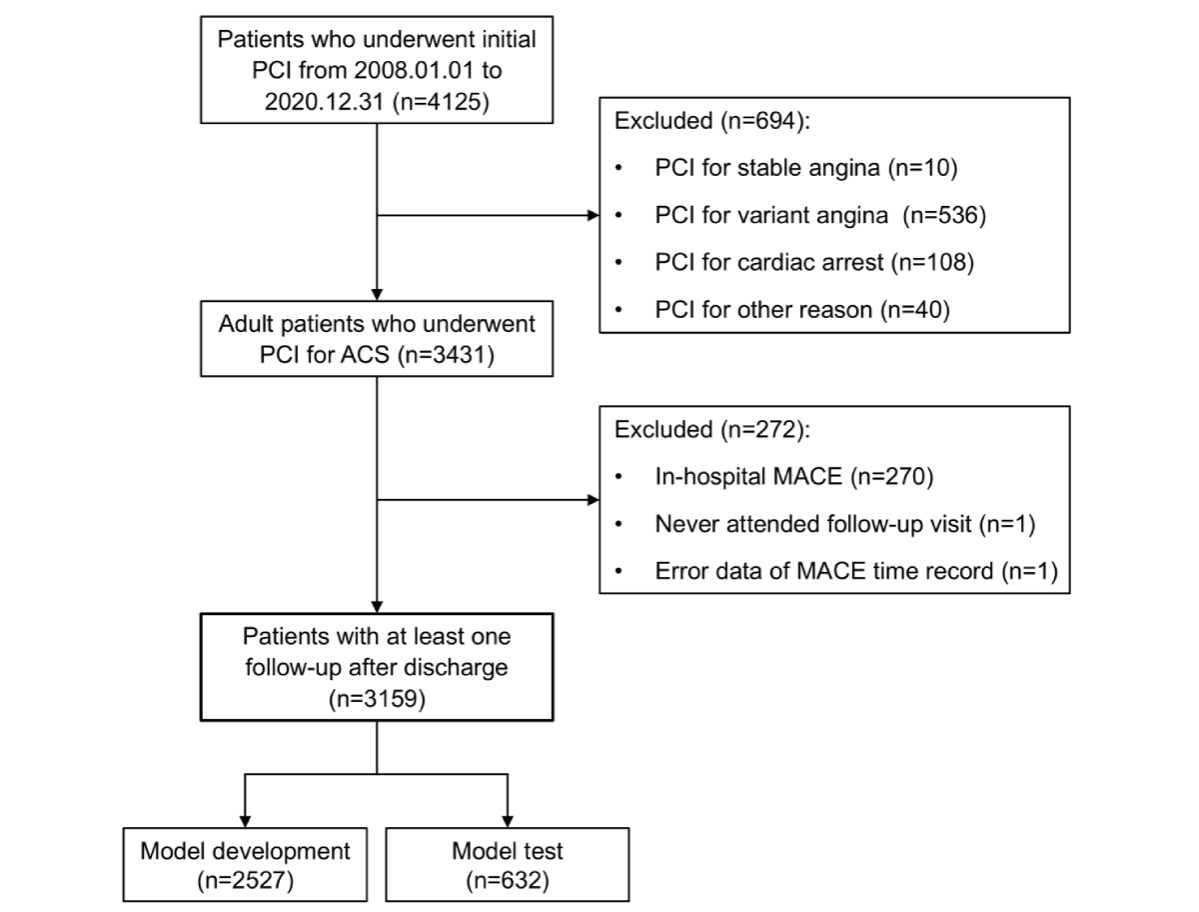
Flowchart of the study population. ACS: acute coronary syndrome; MACE: major adverse cardiovascular events; PCI: percutaneous coronary intervention.

The required sample size was determined on the basis of the number of predictors and the incidence of 1-year MACE, following the established recommendations for prediction model development [28]. An estimated 1-year MACE incidence ranging from 14.8% to 34.4% was considered [14,15]. The minimum required sample size calculated using the lowest event rate (14.8%) to ensure adequate statistical power was 3006 patients. The final analytical cohort was composed of 3159 patients who satisfied all eligibility criteria (Figure 1).

### Data Collection

Based on an extensive review of the literature and the clinical expertise of the cardiology and cardiovascular nursing team participating in the study, 54 variables were selected as potential predictors of MACE. These variables covered a broad range of patient characteristics, including demographic variables, lifestyle-related variables, comorbidities, PCI-related variables, laboratory tests, medication status, and medication adherence. The predictor variables and measurement methods are described in detail in Multimedia Appendix 3.

### Predictors

The final set of predictors included 54 variables classified into 7 categories: demographic variables (eg, age, sex, and insurance type), lifestyle-related variables (eg, smoking status and alcohol consumption), comorbidities (eg, presence of hypertension, renal impairment, or diabetes mellitus), PCI-related variables (eg, contrast volume, catheterization status, and left ventricular ejection fraction), laboratory tests (eg, troponin I and glomerular filtration rate [GFR]), medication status (eg, use of antiplatelets, statins, and beta blockers), and medication adherence indicators (eg, medication regimen complexity index [MRCI]).

The nutrition risk index was used to assess nutritional status, where low values indicate a high nutritional risk [29]. The catheterization status was categorized as elective, urgent, emergent, or salvage procedures based on the clinical context [30]. The MRCI was used to quantify the complexity of each patient’s medication regimen; high scores reflected increased regimen complexity and potentially poor medication adherence [31,32]. Changes in the MRCI between admission and discharge (MRCI changed) were evaluated as a dynamic indicator of medication adjustment during hospitalization. Laboratory test values were collected on the basis of the most unfavorable results on the day of admission, reflecting the worst clinical status likely to influence outcomes. A complete list of all predictors and their detailed definitions is available in Multimedia Appendix 3.

### Outcome Variable: MACE

The primary outcome of this study was the time to the first occurrence of MACE after hospital discharge as identified through EMRs. MACE was defined as a composite outcome comprising cardiovascular death, myocardial infarction, stroke, or revascularization according to standardized definitions from prior consensus guidelines [4,33]. The final date for data collection was December 31, 2021, and survival time was calculated from the date of hospital discharge.

### Statistical Analysis

Means and SDs were used to summarize continuous data; categorical data were reported as counts and percentages. Groups were statistically compared using 2-tailed *t* tests and chi-square tests.

For the time-to-event analysis, patients who did not experience MACE during follow-up were treated as censored cases, and MACE occurrence was coded as a binary outcome (1=event, 0=no event). Statistical analyses were performed using SPSS (version 25.0; IBM Corp), and the time-to-event ML model was developed in Python (version 3.9.13; Python Software Foundation). All tests were 2-tailed, and the significance level was set at *P*<.05.

### Model Development

The valid ranges of all variables were reviewed and confirmed by the cardiology and cardiovascular nursing team involved in this study. Missing values were assumed to follow a missing-at-random pattern and handled using standard imputation techniques; specifically, the mode was used for binary and categorical variables, and the mean was used for continuous variables. Continuous variables were normalized using minimum–maximum scaling, and categorical variables were processed using one-hot encoding.

The dataset was stratified by MACE occurrence and randomly split into a training set (80%) and a test set (20%) to preserve the event-to-censoring ratio across sets. Five-fold cross-validation within the training set was applied to evaluate model discrimination and calibration and to minimize the risk of overfitting. Among the time-to-event ML models evaluated, Survival Quilts served as the primary algorithm. Survival Quilts is an open-source AutoML framework (implemented from Survival Quilts GitHub [34]) that constructs an ensemble of baseline time-to-event models and adaptively adjusts their weights across time horizons to optimize discriminative performance while maintaining calibration constraints [24].

In total, six time-to-event ML models were developed and compared: Survival Quilts, ensemble-based survival models including Random Survival Forest and CoxBoost (implemented with *scikit-survival* v0.23.0); parametric accelerated failure time models including LogNormal and Weibull (implemented with *lifelines* v0.30.0), and the Cox proportional hazards model (implemented with *scikit-survival* v0.23.0).

Model development was conducted using the training set, with 20% of this training data reserved as a validation subset for hyperparameter optimization. Hyperparameters were tuned through an exhaustive grid search. For ensemble models (Random Survival Forest and CoxBoost), the number of estimators was varied across (50, 100, 200, 300, 400, 500). For accelerated failure time models (LogNormal and Weibull) and the Cox model, 10 regularization coefficients logarithmically spaced between 10e-3 and 1 were tested. For Survival Quilts, time horizons were defined as (30, 60, 90, 180, 270, 365), while all other parameters were set to default values. Random seeds were fixed (seed=1234) across all analyses to ensure reproducibility.

### Model Performance

The performance of the time-to-event ML models was evaluated on the basis of discrimination and calibration, which are essential factors for assessing the clinical use of time-to-event ML models [24]. Model performance was examined using the metrics tailored for right-censored data: the time-dependent concordance index (C-index) for discrimination and the time-dependent Brier score for overall prediction accuracy. The time-dependent C-index measures the effectiveness of the model in distinguishing individual risks at different time points, particularly in the presence of censored observations [35]. The time-dependent Brier score assesses the accuracy of predicted survival probabilities by comparing them with the actual distribution of observed events across multiple time points [36].

Permutation-based variable importance scores were calculated to assess the contribution of individual predictors to model performance. Through this method, the decrease in model performance is quantified when the values of a specific variable are randomly permuted, thereby disrupting its relationship with the outcome [37]. The greater the reduction in model performance, the greater the importance of that variable. The time-dependent C-index was used as the reference metric for assessing the effect of each permutation; a large drop indicated a strong influence on the model’s discriminative power.

Partial dependence plots were used to show the effect of changes in each predictor on the model’s predicted risk. For continuous variables, predictor values varied from minimum to maximum, and the corresponding changes in the predicted risk were plotted [25]. For binary variables, predictions were compared between 0 and 1. For categorical variables, predictions were calculated for each category.

### Ethical Considerations

The study was conducted in accordance with the ethical principles outlined in the Declaration of Helsinki. This study was approved by the institutional review board of Seoul Metropolitan Government-Seoul National University Boramae Medical Center, Seoul, Korea (IRB No 30-2024-31).

## Results

### Baseline Characteristics

The baseline characteristics of the study participants are summarized in Multimedia Appendix 4. The median follow-up duration for the total cohort (N=3159) was 3.8 (IQR 1.6-6.7) years; during follow-up, 626 (19.8%) patients experienced MACE. The mean age of the total patient population was 66.8 years, and 2084 (66%) patients were male. The MACE group was significantly older than the censored group (68.33, SD 11.29 vs 66.42, SD 11.77 years; *P*<.001).

Comorbidities were more common in the MACE group than in the censored group. The prevalence of hypertension was significantly higher in the MACE group (420/626, 67.1%) than in the censored group (1573/2533, 62.1%; *P*=.02). Similarly, higher proportions were observed for peripheral artery disease (23/626, 3.7% vs 41/2533, 1.6%; *P*=.002), atrial fibrillation (39/626, 6.2% vs 90/2533, 3.6%; *P*=.004), heart failure (50/626, 8% vs 81/2533, 3.2%; *P*<.001), chronic kidney disease (63/626, 10.1% vs 126/2533 5.0%; *P*<.001), and dialysis-dependent renal failure (32/626, 5.1% vs 49/2533, 1.9%; *P*<.001). The prevalence of diabetes mellitus differed significantly between the MACE and censored groups (*χ*²_3_=39.7, *P*<.001). Patients without diabetes were less common in the MACE group (361/626, 57.7%) than in the censored group (1718/2533, 67.8%).

Unstable angina was the most frequent ACS subtype (1703/3159, 53.9%) among patients who underwent PCI, and 50.4% (1592/3159) were admitted via the emergency department. Overall, 40.5% (1281/3159) of patients had three-vessel disease, and 7.3% (230/3159) had left main disease. The MACE group received a significantly larger contrast volume during PCI than the censored group (300.66, SD 140.38 mL vs 256.10, SD 125.98 mL; *P*<.001). The severity of CAD was also greater in the MACE group, with three-vessel disease observed in 53.7% (336/626) patients compared with 37.3% (945/2533) patients in the censored group (*P*<.001).

In general, patients who experienced MACE had lower lipid profiles and higher C-reactive protein levels than those without MACE. GFR was also lower in the MACE group (70.68, SD 28.73 mL/min/1.73 m² vs 77.25, SD 26.26 mL/min/1.73 m²; *P*<.001). Among baseline medications, the most frequently prescribed were antiplatelets (2932/3159, 92.8%), followed by nitrates (1992/3159, 63.1%) and statins (1706/3159, 54%). MRCI at discharge was significantly higher in the MACE group (26.14, SD 12.68 vs 24.91, SD 11.20; *P*=.03).

**Figure 2:**
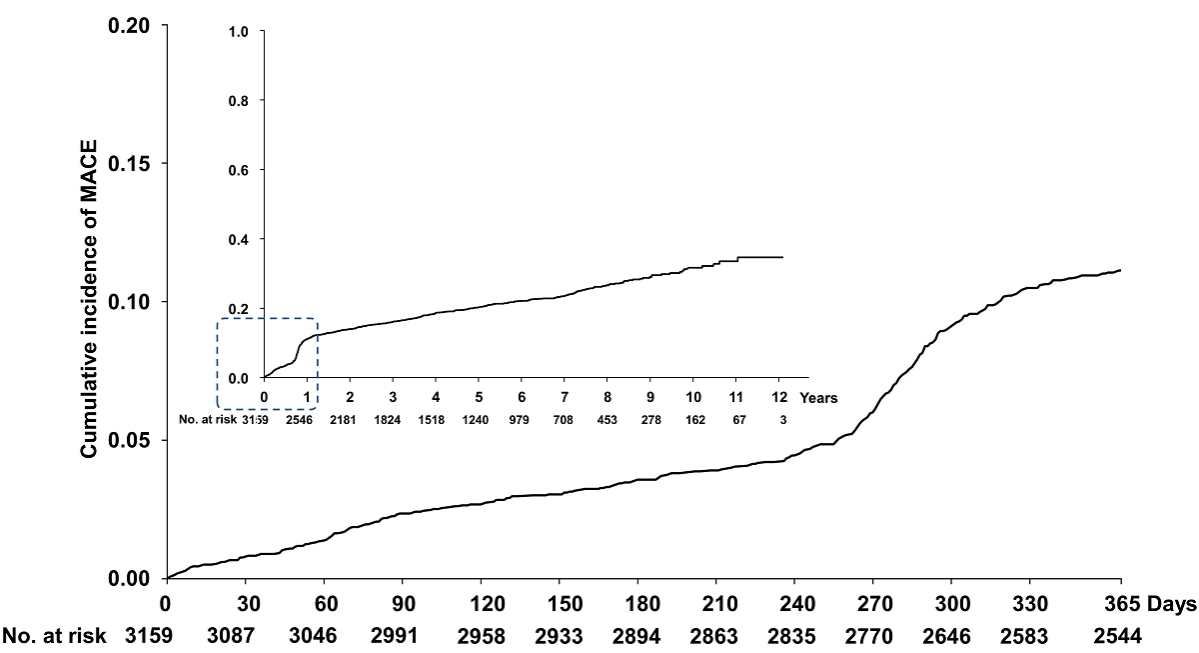
Incidence of major adverse cardiac events (MACE) after percutaneous coronary intervention (PCI) in patients with acute coronary syndrome ACS.

Figure 2 shows the Kaplan–Meier curve of the cumulative MACE rate after hospital discharge. The main graph presents the 1-year survival curve. Within 1 year, the incidence of MACE increased gradually over time; after approximately 270 days, it increased more sharply. The cumulative MACE rates were 0.8%, 3.6%, and 11.1% after 30 days, 180 days, and 1 year, respectively. Our analysis primarily focused on the first year following discharge because most MACE occur, and early intervention is likely to be the most effective during this period.

### Model Performance

**Figure 3:**
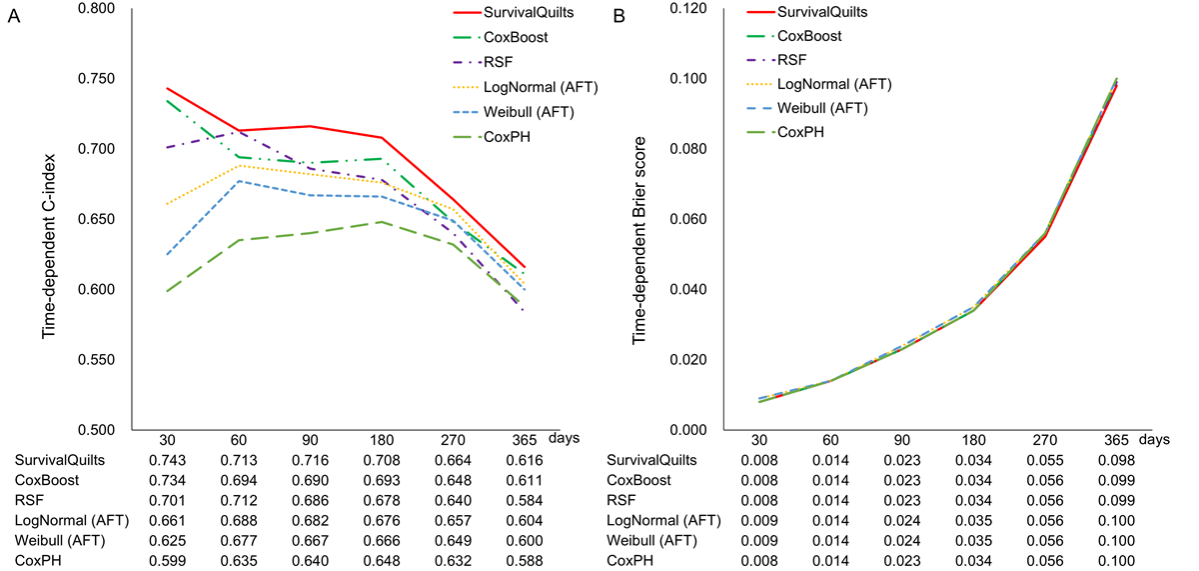
Comparison of the time-dependent model performance for major adverse cardiac events (MACE) prediction. (A) Time-dependent concordance index (C-index) from 30 days to 365 days. (B) Time-dependent Brier scores from 30 days to 365 days. AFT: accelerated failure time; CoxPH: Cox proportional hazards model; RSF: Random Survival Forest.

We evaluated the predictive performance of the models for MACE by using the time-dependent C-index and the time-dependent Brier score (Figure 3). Figure 3A presents the time-dependent C-index of multiple models, including Survival Quilts, CoxBoost, Random Survival Forest, LogNormal, Weibull, and Cox proportional hazards model. We compared the model performance on days 30, 60, 90, 180, 270, and 365. Although the time-dependent C-index values decreased over time across all models, the Survival Quilts model consistently demonstrated the highest discriminative performance at each time point. It also showed the highest C-index of 0.743 on day 30, but this index gradually declined to 0.616 on day 365. Figure 3B displays the time-dependent Brier scores for the same models. Overall, the Brier scores increased over time. All models demonstrated comparable and stable Brier scores in the observed time points.

Among the 6 time-to-event ML models compared, Survival Quilts exhibited the best overall performance based on the time-dependent C-index and Brier score. Therefore, Survival Quilts was defined as the best model in this study, and subsequent analyses, including predictor importance and time-varying risk contribution, were conducted using this model. A web-based demonstrator of the best-performing model is available at Streamlit [38], where individualized MACE risk predictions based on the Survival Quilts algorithm can be interactively explored.

### Variable Importance in the Best Model

Table 1 presents the permutation-based variable importance of the best model at different time points. At 30 days post discharge, contrast volume emerged as the strongest predictor, followed by age and the MRCI at discharge. On day 60, the most important predictor was CAD severity, followed by GFR. Conversely, the importance of age and MRCI at discharge decreased. After day 60, CAD severity and GFR remained the most important predictors throughout the follow-up.

**Table 1:** Top 10 predictor variables for major adverse cardiovascular events (MACE) over time in the best machine learning model.

Rank	30 days	60 days	90 days	180 days	270 days	365 days
1	Contrast volume	CAD^a^ severity	CAD severity	CAD severity	CAD severity	CAD severity
2	Age	GFR^b^	GFR	CRP^c^	GFR	GFR
3	MRCI^d^ on discharge	CRP	Uric acid	GFR	IABP^e^ used	Uric acid
4	LVEF^f^	Uric acid	Age	Uric acid	Atrial fibrillation	IABP used
5	LDL^g^	LDL	CRP	Contrast volume	Uric acid	Creatinine
6	Severity CAD	Creatinine	Atrial fibrillation	IABP used	LVEF	LVEF
7	Uric acid	LVEF	LVEF	Atrial fibrillation	Insurance type	Total cholesterol
8	Fluoro duration	Age	IABP used	LVEF	Contrast volume	Dialysis
9	CRP	Dialysis	Insurance type	HbA_1c_	Dialysis	Fluoro duration
10	Hemoglobin	MRCI on discharge	Creatinine	Insurance type	Hemoglobin	Insurance type

^a^CAD: coronary artery disease.

^b^GFR: glomerular filtration rate.

^c^CRP: C-reactive protein.

^d^MRCI: medication regimen complexity index.

^e^IABP: intra-aortic balloon pump.

^f^LVEF: left ventricular ejection fraction.

^g^LDL: low density lipoprotein.

**Figure 4:**
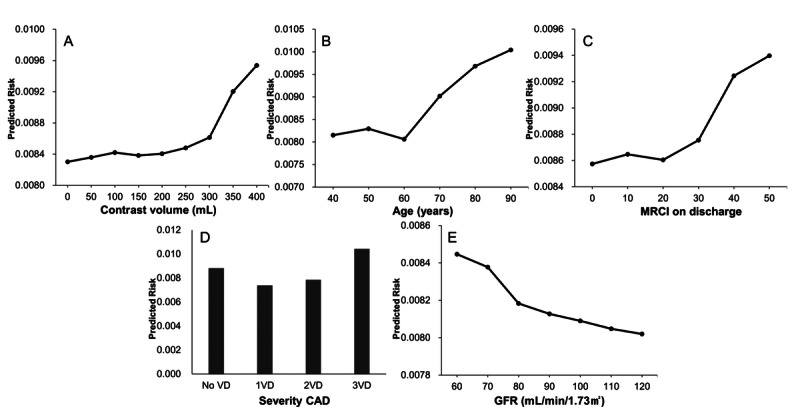
Partial dependence plots of key predictors of major adverse cardiovascular events (MACE) based on the best machine learning (ML) model. (A) Contrast volume and predicted risk of MACE. (B) Age and predicted risk of MACE. (C) The medication regimen complexity index (MRCI) at discharge and predicted risk of MACE. (D) Severity of coronary artery disease (CAD) and predicted MACE. (E) The glomerular filtration rate (GFR) and predicted risk of MACE. VD: vessel disease.

Figure 4 shows the partial dependence plots of the top predictors in the best model: contrast volume, age, MRCI at discharge, CAD severity, and GFR. An increase in contrast volume was associated with a high risk of MACE occurrence, particularly when the contrast volume exceeded 300 mL (Figure 4A). Age showed similar predicted risks of MACE occurrence among patients aged 40-49 years and 60-69 years; however, the risk increased sharply in patients aged >60 years (Figure 4B). The MRCI at discharge minimally influenced the MACE occurrence risk up to a score of 20, but the risk progressively increased above this threshold; it showed a steep rise beyond 30 points (Figure 4C). The MACE occurrence risk of patients with three-vessel disease was higher than that of patients with single- or two-vessel disease (Figure 4D). A high GFR was associated with a low MACE occurrence risk, while the risk increased sharply in patients with GFR below 80 mL/min/1.73 m^2^ (Figure 4E).

**Figure 5:**
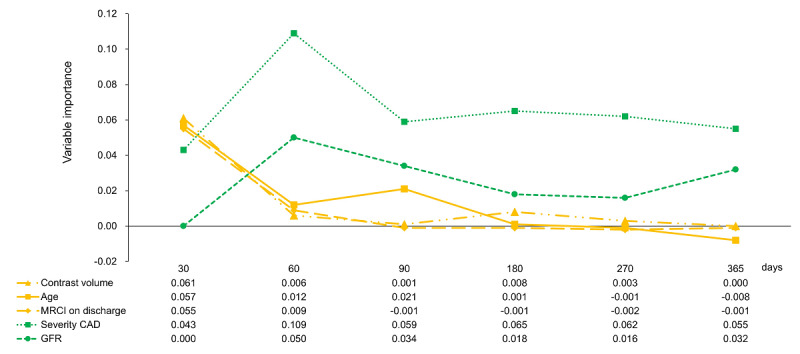
Temporal variation in the importance of key variables in the best machine learning (ML) model. CAD: coronary artery disease; GFR: glomerular filtration rate; MRCI: medication regimen complexity index.

Figure 5 illustrates the time-varying patterns of variable importance for the key predictors based on permutation analysis. Contrast volume, age, and MRCI at discharge showed the highest importance on day 30, but they gradually declined over time. Conversely, CAD severity and GFR became more important after day 60 and maintained high importance throughout the follow-up.

## Discussion

### Overview

We identified 5 key predictors, namely, contrast volume, age, medication adherence (measured by MRCI at discharge), severity of CAD, and GFR, which showed time-varying importance in predicting MACE after PCI in patients with ACS. The risk of MACE occurrence exhibited nonlinear patterns; specifically, abrupt changes were observed at specific thresholds rather than a steady increase or decrease. Furthermore, the temporal patterns of predictor importance revealed 2 distinct trends. First, contrast volume, age, and medication adherence were the most influential predictors within the first 30 days after hospital discharge; however, their importance diminished over time. Our findings highlighted the importance of optimizing care during the early postdischarge period to prevent early adverse events. Second, CAD severity and renal function emerged as the dominant predictors after 60 days and remained consistently important throughout the follow-up. These insights were obtained through the application of a time-to-event ML framework. This framework could be used to identify nonlinear risk patterns and temporal changes in predictor importance, which are difficult to achieve using traditional statistical methods. By focusing on the first year after discharge, the period of the highest clinical vulnerability could be determined. Thus, actionable insights into timely intervention could be provided.

### Early Hospital Discharge Period: Within 30 Days

In this study, the risk of MACE increased sharply when the contrast volume exceeded 300 mL. Contrast volume was identified as the most important variable in the early hospital discharge period, particularly within the first 30 days. The sharp increase in MACE risk above the 300 mL threshold suggests a nonlinear relationship; thus, contrast volume was an important early determinant of MACE after PCI. This finding highlighted the relevance of minimizing contrast exposure during the procedure to reduce MACE early after hospital discharge.

Our results were consistent with previous studies reporting the association between a higher contrast volume and the increased risk of contrast-induced nephropathy and MACE [39,40]. Saito and Kobayashi [41] examined patients with ACS who received PCI and found that those treated for multivessel disease received a higher average contrast volume (295 mL) than those with single-vessel disease (180 mL); they also exhibited a significantly increased MACE incidence. Similarly, Yao et al [40] reported that contrast volumes exceeding 300 mL are a strong independent risk factor for kidney injury, which in turn increases MACE incidence. Notably, Ng et al [39] showed that contrast-induced nephropathy is associated with an approximately 82% increase in MACE risk within the first 30 days after discharge; however, they also demonstrated that this risk decreased to 13% between 30 days and 1 year and was no longer statistically significant beyond 12 months.

This time-varying importance of contrast volume may be explained by the transient effect of contrast-induced renal injury occurring in the early post-PCI period. The early increase in MACE risk is attributed to the occurrence of acute kidney injury shortly after PCI; however, its effect decreases as renal function stabilizes over time [42]. Therefore, targeted efforts to minimize contrast use are crucial, particularly in the periprocedural and early period after hospital discharge.

From a clinical perspective, the health care team should work collaboratively to reduce contrast use during PCI. After PCI, information regarding contrast volume should be clearly communicated to the ward team to facilitate ongoing monitoring of renal function, particularly in patients who receive more than 300 mL of contrast and may require closer follow-up. Previous quality improvement initiatives established that interdisciplinary collaboration could reduce the incidence of contrast-induced renal impairment by up to 21% [43]. In patients who had ACS treated with PCI and received a large contrast volume, outcome improvement may depend on effective interdisciplinary communication and coordinated care efforts. In addition, emerging technologies such as the dynamic coronary roadmap, which provides real-time vascular guidance without the need for repeated contrast injections, show potential for applications in further reducing contrast volume during PCI [44]. The adoption of such innovations in clinical practice may help prevent contrast-related complications and reduce the incidence of MACE following PCI.

In this study, age was identified as an important predictor of MACE, particularly in the early period after hospital discharge. The risk of MACE remained relatively stable among patients aged 40-60 years but increased sharply in those older than 60 years. The importance of age as a predictor was highest at 30 days after discharge and decreased over time. This finding suggested that older patients are at a greater risk of early MACE following PCI and may benefit from closer monitoring during this period. These results were consistent with prior evidence suggesting that age is a recognized contributor to an increased MACE risk [45-47]. The increased vulnerability associated with advancing age may be explained by the cumulative effects of vascular degeneration, increased prevalence of comorbidities, and reduced medication adherence [45,47]. Helber et al [48] reported that patients aged 75 years and older who underwent PCI for ST-elevation myocardial infarction (STEMI) have higher risks of in-hospital complications, including bleeding, cardiogenic shock, and mortality, than younger patients. However, Chen et al [49] found that MACE incidence does not significantly differ between older and younger groups at 6 months post-PCI. This result supports the interpretation that the influence of age on MACE risk diminishes over time as the effect of acute complications decreases. These findings indicate that management immediately after the hospital discharge of older patients with ACS subjected to PCI may help reduce the risk of early MACE through enhanced surveillance and tailored interventions.

A key finding of this study is that lower medication adherence, as reflected by higher MRCI at discharge, was notably related to a higher likelihood of MACE occurrence, particularly during the early period after hospital discharge. In our cohort, the mean MRCI score at discharge was approximately 25, which typically indicated that patients were prescribed 2 or more additional medications beyond standard PCI-related medications because of comorbid conditions.

Further subgroup analysis based on MRCI component scores, namely, dosage forms, dosing frequency, and additional user instructions, revealed that the patients with MRCI scores of 20, 25, and 30 exhibited a progressively higher MACE risk (Multimedia Appendix 5). Differences in dosage forms were significant only between the lowest (20) and highest (30) score groups; conversely, dosing frequency and additional user instructions significantly differed across all 3 groups. These findings indicated that all components of medication adherence—namely, dosage forms, dosing frequency, and additional user instructions—play a meaningful role in influencing MACE risk; therefore, comprehensive strategies should be developed to simplify medication regimens. This finding is consistent with earlier research, which indicated that the increased regimen complexity reduces medication adherence and consequently raises MACE risk [50,51]. In this study, the predictive importance of medication adherence was also the highest within the first 30 days during the early period after hospital discharge and declined thereafter. Therefore, early intervention strategies should be developed to simplify medication regimens and improve adherence, particularly during the early period after hospital discharge, to reduce the risk of MACE.

### Later Hospital Discharge Period: From 60 Days to 1 Year

In this study, CAD severity and renal function, measured by GFR, emerged as the most important predictors of MACE during the later period after hospital discharge, particularly beyond 60 days. Patients with more severe CAD, especially those with three-vessel disease, showed a consistently higher risk of MACE over time. Similarly, reduced GFR was related to an increased risk of MACE, independent of acute kidney injury related to contrast exposure.

These findings were consistent with prior research, which demonstrated that the extent of CAD reflects the burden of atherosclerosis, which increases the likelihood of new lesion development or restenosis over time [52]. Three-vessel disease has been well established as a predictor of long-term MACE because of factors such as persistent myocardial ischemia, plaque instability, and progression of atherosclerosis [4,45,47]. Regarding renal function, our findings highlight that lower GFR remained a significant independent risk factor for MACE even beyond the early period of PCI. This finding was consistent with previous studies showing that reduced GFR, reflecting chronic kidney dysfunction, independently contributes to increased MACE regardless of contrast-induced acute kidney injury [53]. Decreased GFR reflects the progression of chronic kidney disease, which is closely associated with the increased long-term MACE incidence [54].

### Time-to-Event ML for Risk Prediction

The application of a time-to-event ML framework enabled the identification of time-varying predictor importance and nonlinear risk patterns for MACE following PCI in patients with ACS. Through this approach, risk estimation can be performed to reflect the changes over time at different hospital discharge periods, which is difficult to achieve using traditional statistical models, such as the Cox proportional hazards model. These findings were consistent with previous studies that demonstrated the utility of time-to-event ML models in various clinical contexts. For example, Choi et al [25] applied a time-to-event ML approach to patients who experienced cardiac arrest and showed that temporal changes in predictor importance can guide the optimal timing of reperfusion therapies. Similarly, Lee et al [24] highlighted that incorporating time-to-event ML improves prediction accuracy and supports more informed clinical decision-making.

The ability of time-to-event ML models to determine changes in risk over time and nonlinear relationships provides meaningful advantages in long-term risk prediction. In patients with ACS undergoing PCI, this approach offers the potential to inform the timing of tailored interventions based on changes in risk over time. Future studies are warranted to validate these methods in larger and more diverse patient populations and to explore their integration into real-time clinical decision support systems.

### Strengths and Implications

This study has several key strengths. First, we developed a ML model specifically for patients with ACS undergoing PCI; thus, MACE could be more accurately predicted by incorporating time-to-event information. Second, we identified nonlinear risk patterns and specific thresholds for key predictors using permutation-based variable importance and partial dependence plots, providing deeper insights into risk dynamics. Third, our approach could estimate how risk changes over time, which can support the planning of tailored interventions at different time points after hospital discharge.

### Limitations

This study is subject to several limitations. First, because the model was developed and internally validated using data from a single center, there remains a potential risk of overfitting and optimistic performance estimates despite the use of 5-fold cross-validation. External validation using multicenter data is required to confirm generalizability. Second, missing values were handled under a missing-at-random assumption using simple imputation. However, this approach may underestimate variability and does not fully capture the uncertainty associated with missing data. More advanced strategies, such as multiple imputation or the k-fold cross validation, could be explored in future work to reduce potential bias. Third, as this study relied on EMR data, the available predictors were limited; some potential risk factors, such as psychosocial variables including stress, were also not considered. Fourth, all patient data were collected prior to PCI, and laboratory values were represented using either maximum values or averaged results from multiple measurements. Thus, temporal changes in patient condition after PCI were not reflected in the model. Fifth, medication adherence was indirectly assessed using the MRCI, which measures regimen complexity rather than actual adherence behavior. Although MRCI demonstrated good reliability in this population, future studies should incorporate the direct measures of medication adherence to improve predictive accuracy. Sixth, although the study identified a nonlinear relationship between contrast volume and MACE risk, particularly beyond 300 mL, factors such as body weight and renal function ratio were not considered; results should be carefully interpreted, and further investigation should be performed. Finally, because of the nature of secondary data analysis, there may have been data entry errors or missing values, which could have influenced the results. Despite these limitations, this study provides meaningful contributions by demonstrating the utility of time-to-event ML in predicting MACE in patients with ACS undergoing PCI and by identifying key risk factors that may inform clinical practice.

### Conclusions

We developed a time-to-event ML model to predict MACE in patients with ACS undergoing PCI. This model incorporated time-dependent risk estimation during the entire follow-up; thus, nonlinear risk patterns and temporal changes in predictor importance could be identified, thereby enhancing interpretability and clinical applicability. Notably, contrast volume, age, and medication adherence exhibited the greatest influence within the first 30 days after hospital discharge; conversely, the severity of CAD and GFR became more influential in the later period, particularly beyond 60 days. Although the model was trained using full follow-up data, our findings highlighted that the first year after discharge was a critical window for intervention. During this period, targeted strategies, such as minimizing contrast use, closely monitoring renal function, and improving medication adherence, might provide the most clinical benefit. These insights might help health care providers implement time-informed, personalized strategies to improve patient outcomes.

## Appendix

Multimedia Appendix 1 TRIPOD+AI checklist.

Multimedia Appendix 2 STROBE statement.

Multimedia Appendix 3 Predictors used in the time-to-event machine learning (ML) model.

Multimedia Appendix 4 Baseline characteristics of patients who underwent percutaneous coronary intervention (PCI) for acute coronary syndrome (ACS; N= 3159).

Multimedia Appendix 5 Three medication regimen complexity index (MRCI) components across score-based subgroups and the total cohort.

## Abbreviations

ACS: acute coronary syndrome
CAD: coronary artery disease
C-index: concordance index
EMR: electronic medical record
GFR: glomerular filtration rate
MACE: major adverse cardiovascular events
ML: machine learning
MRCI: medication regimen complexity index
PCI: percutaneous coronary intervention
STEMI: ST-elevation myocardial infarction
STROBE: Strengthening the Reporting of Observational Studies in Epidemiology
TRIPOD+AI: Transparent Reporting of a Multivariable Prediction Model for Individual Prognosis or Diagnosis + Artificial Intelligence

## Data Availability

The datasets generated or analyzed during this study are available from the corresponding author on reasonable request.
