# Supplementary figures and images for: Risk Prediction of Major Adverse Cardiovascular Events Within One Year After Percutaneous Coronary Intervention in Patients With Acute Coronary Syndrome: Machine Learning–Based Time-to-Event Analysis
Source: JMIR Med Inform. 2025 Nov 27;13:e81778. doi: 10.2196/81778 (PMC12699253; doi:10.2196/81778)

Multimedia Appendix 1. TRIPOD+AI checklist


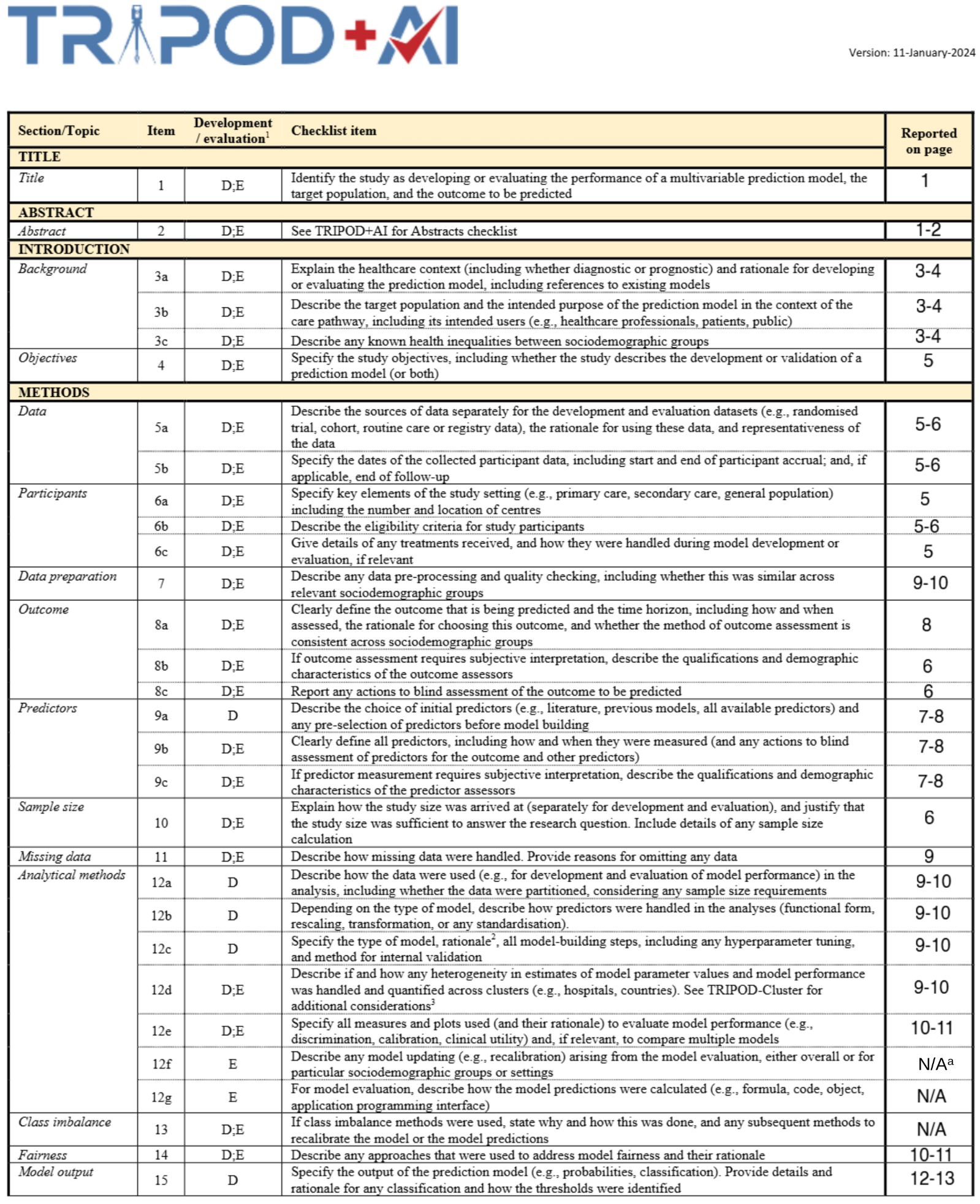


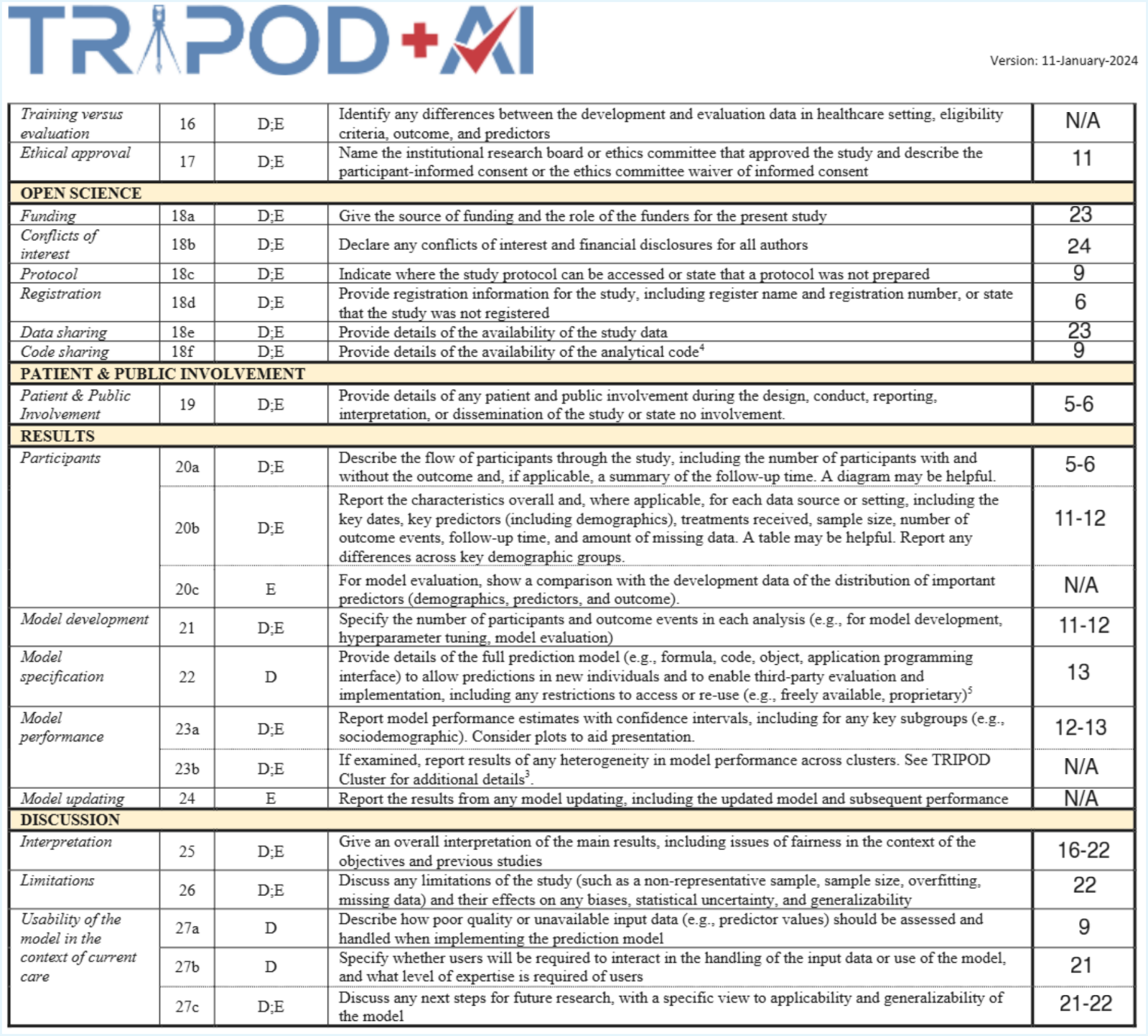


aN/A: not applicable

Supplement: Multimedia Appendix 1 [file medinform_v13i1e81778_app1.docx]
