# Supplementary material for: Risk Prediction of Major Adverse Cardiovascular Events Within One Year After Percutaneous Coronary Intervention in Patients With Acute Coronary Syndrome: Machine Learning–Based Time-to-Event Analysis
Source: JMIR Med Inform. 2025 Nov 27;13:e81778. doi: 10.2196/81778 (PMC12699253; doi:10.2196/81778)
Supplement: Multimedia Appendix 3 [file medinform_v13i1e81778_app3.docx]

Multimedia Appendix 3. Description of predictors used in the time-to-event machine learning model

| Variables | Description | Variable type | Value | Missing rate | Imputation method |
| --- | --- | --- | --- | --- | --- |
| **Demographic variables** |  |  |  |  |  |
| Age, years | Age of the patients | Numeric | Value | 0.0% | N/A^a^ |
| Sex | Sex of the patients | Binary | 0=Female  1=Male | 0.0% | N/A |
| Insurance type | The Republic of Korea’s universal health insurance system includes two main insurance services―national health insurance and medical aid. National Health Insurance, Medical Aid, Other (Automobile insurance and industrial accident compensation insurance) | Category | 0=NHI  1=Medical aid  2=Other | 0.0% | N/A |
| CAD FHx | Family history of coronary artery disease was defined as having a father diagnosed with CAD before the age of 55 or a mother diagnosed with CAD before the age of 65. If either parent met this criterion, the patients had a family history of CAD | Binary | 0=No  1=Yes | 0.0% | N/A |
| **Lifestyle-related variables** |  |  |  |  |  |
| Smoking status | Smoking status (never, ex-smoker, current smoker, unknown | Category | 0=Never  1=Past smoker  2=Current smoker  3=Unknown | 0.0% | N/A |
| Body mass index, kg/ m^2^ | Body mass index of the patients | Numeric | Value | 4.7% | Mean imputation |
| Alcohol consumption | Alcohol consumption status of the patients (None, Mild to moderate, Heavy) | Category | 0=None  1=Mild to moderate  2=Heavy | 70.6% | Mode imputation |
| Nutrition risk index | Nutrition risk index assessing the nutritional risk of the patients | Numeric | Value | 13.4% | Mean imputation |
| **Comorbidity** |  |  |  |  |  |
| Hypertension | History of hypertension | Binary | 0=No  1=Yes | 0.0% | N/A |
| Peripheral artery disease | History of peripheral artery disease | Binary | 0=No  1=Yes | 0.0% | N/A |
| COPD | History of chronic obstructive pulmonary disease | Binary | 0=No  1=Yes | 0.0% | N/A |
| Dyslipidemia | History of dyslipidemia | Binary | 0=No  1=Yes | 0.0% | N/A |
| Heart failure | History of heart failure | Binary | 0=No  1=Yes | 0.0% | N/A |
| Atrial fibrillation | The presence of atrial fibrillation | Binary | 0=No  1=Yes | 0.0% | N/A |
| Chronic kidney disease | History of chronic kidney disease | Binary | 0=No  1=Yes | 0.0% | N/A |
| Dialysis | History of dialysis treatment | Binary | 0=No  1=Yes | 0.0% | N/A |
| Cerebrovascular accident | History of cerebrovascular accident | Binary | 0=No  1=Yes | 0.0% | N/A |
| Diabetes mellitus | History of diabetes mellitus | Category | 0=None  1=Without medication  2=With OHA  3=With insulin | 0.0% | N/A |
| **PCI-related variables** |  |  |  |  |  |
| Admission route | Route of hospital admission for the patients (outpatient department, emergency department, non-acute transfer, acute transfer) | Category | 0=Outpatients department  1=Emergency department  2=Non-acute transfer  3=Acute transfer | 0.1% | Mode imputation |
| Fluoro duration | Duration of fluoroscopy exposure during percutaneous coronary intervention | Numeric | Value | 2.9% | Mean imputation |
| Contrast volume | Used contrast volume during percutaneous coronary intervention | Numeric | Value | 0.0% | N/A |
| Catheterization status | Urgency of the percutaneous coronary intervention procedure, classified as elective, urgent, emergent, or salvage | Category | 0=Elective  1=Urgent  2=Emergent  3=Salvage | 0.1% | Mode imputation |
| Access route | Puncture site used for performing PCI (radial, femoral, other arteries) | Category | 0=Radial artery  1=Femoral artery  2=Other artery | 0.0% | N/A |
| IABP used | Intra-aortic balloon pump was performed during percutaneous coronary intervention | Binary | 0=No  1=Yes | 0.0% | N/A |
| LVEF, % | Left ventricular ejection fraction via echocardiogram | Numeric | Value | 49.5% | Mean imputation |
| RWMA | The presence of regional wall motion abnormality | Binary | 0=No  1=Yes | 0.0% | N/A |
| ST segment in EKG | ST segment changed in electrocardiogram | Category | 0=Normal  1=ST depression  2=ST elevation  3=Nonspecific | 0.0% | N/A |
| LM desease | The presence of left main disease | Binary | 0=No  1=Yes | 0.0% | N/A |
| Severity CAD | Severity of coronary artery disease in the patients | Category | 0=No vessle disease  1=1 vessle disease  2=2 vessle disease  3=3 vessle disease | 0.0% | N/A |
| TIMI grade flow | Thrombolysis in myocardial infarction grade flow assessing blood flow through coronary arteries | Category | 0=Grade 0  1=Grade 1  2=Grade 2  3=Grade 3 | 1.5% | Mode imputation |
| PCI vessel | Target vessel for percutaneous coronary intervention | Category | 0=Left main  1=LAD  2=Left circumflex artery  3=Right coronary artery | 0.0% | N/A |
| **Laboratory test** |  |  |  |  |  |
| Total cholesterol, mg/dL | Serum total cholesterol levels | Numeric | Value | 9.4% | Mean imputation |
| HDL, mg/dL | Serum high-density lipoprotein cholesterol levels | Numeric | Value | 53.6% | Mean imputation |
| LDL, mg/dL | Serum low-density lipoprotein cholesterol levels | Numeric | Value | 53.4% | Mean imputation |
| Triglyceride, mg/dL | Serum triglyceride levels | Numeric | Value | 53.8% | Mean imputation |
| CKMB, ng/ml | Serum Creatine Kinase-MB levels | Numeric | Value | 43.7% | Mean imputation |
| Troponin I, ng/ml | Serum Troponin I levels | Numeric | Value | 54.4% | Mean imputation |
| Creatinine, mg/dL | Serum creatinine levels | Numeric | Value | 2.8% | Mean imputation |
| GFR, ml/min/1.73 m^2^ | Glomerular filtration rate calculated by the modification of diet in renal disease formula | Numeric | Value | 2.9% | Mean imputation |
| HbA1c, % | Serum hemoglobin A1c levels | Numeric | Value | 65.3% | Mean imputation |
| Uric acid, mg/dL | Serum uric acid levels | Numeric | Value | 17.3% | Mean imputation |
| Hemoglobin, g/dL | Serum hemoglobin levels | Numeric | Value | 1.7% | Mean imputation |
| CRP, mg/dL | Serum C-reactive protein levels | Numeric | Value | 34.6% | Mean imputation |
| Albumin | Serum albumin levels | Numeric | Value | 8.7% | Mean imputation |
| **Medication** |  |  |  |  |  |
| Antiplatelets | Use of antiplatelet medications | Binary | 0=No  1=Yes | 0.0% | N/A |
| GpIIb/IIIa inhibitors | Use of glycoprotein IIb/IIIa inhibitors | Binary | 0=No  1=Yes | 0.0% | N/A |
| Statins | Use of statin medications | Binary | 0=No  1=Yes | 0.0% | N/A |
| ACEis | Use of angiotensin-converting enzyme inhibitors | Binary | 0=No  1=Yes | 0.0% | N/A |
| ARBs | Use of angiotensin II receptor blockers | Binary | 0=No  1=Yes | 0.0% | N/A |
| Beta blockers | Use of beta-blocker medications | Binary | 0=No  1=Yes | 0.0% | N/A |
| CCBs | Use of calcium channel blockers | Binary | 0=No  1=Yes | 0.0% | N/A |
| Nitrates | Use of nitrate medications | Binary | 0=No  1=Yes | 0.0% | N/A |
| **Medication adherence** |  |  |  |  |  |
| MRCI on discharge | Medication regimen complexity index at the time of discharge | Numeric | Value | 0.0% | N/A |
| MRCI changed | Change in the Medication regimen Complexity index between admission and discharge | Numeric | Value | 0.0% | N/A |

^a^N/A: not applicable
