# Supplementary material for: Risk Prediction of Major Adverse Cardiovascular Events Within One Year After Percutaneous Coronary Intervention in Patients With Acute Coronary Syndrome: Machine Learning–Based Time-to-Event Analysis
Source: JMIR Med Inform. 2025 Nov 27;13:e81778. doi: 10.2196/81778 (PMC12699253; doi:10.2196/81778)
Supplement: Multimedia Appendix 4 [file medinform_v13i1e81778_app4.docx]

Multimedia Appendix 4. Baseline characteristics of patients underwent PCI^a^ for ACS^b^ (N=3159).

| Characteristics | | | Total  (N=3159) | MACE^c^  (n=626) | Censored  (n=2533) | *P* value |
| --- | --- | --- | --- | --- | --- | --- |
| **Demographic variables** | | |  |  |  |  |
| Age (years), mean (SD^d^) | | | 66.80 (11.70) | 68.33 (11.29) | 66.42 (11.77) | <.001 |
| Sex, n (%) | | |  |  |  |  |
|  | Male | | 2084 (66.0) | 417 (66.6) | 1667 (65.8) | .74 |
|  | Female | | 1075 (34.0) | 209 (33.4) | 866 (34.2) |  |
| Insurance type, n (%) | | |  |  |  |  |
|  | National health insurance | | 2644 (83.7) | 493 (78.8) | 2151 (84.9) | <.001 |
|  | Medical aid | | 436 (13.8) | 115 (18.4) | 321 (12.7) |  |
|  | Other | | 79 (2.5) | 18 (2.9) | 61 (2.4) |  |
| Family history of CAD^e^, n (%) | | | 326 (10.3) | 71 (11.3) | 255 (10.1) | .39 |
| **Lifestyle-related variables** | | |  |  |  |  |
| Smoking status, n (%) | | |  |  |  |  |
|  | Never | | 1264 (40.0) | 245 (39.1) | 1019 (40.2) | .97 |
|  | Ex-smoker | | 815 (25.8) | 163 (26.0) | 652 (25.7) |  |
|  | Current | | 952 (30.1) | 192 (30.7) | 760 (30.0) |  |
|  | Unknown | | 128 (4.1) | 26 (4.2) | 102 (4.0) |  |
| Body mass index (kg/m^2^), mean (SD) | | | 24.50 (3.43) | 24.61 (3.39) | 24.48 (3.44) | .54 |
| Alcohol consumption, n (%) | | |  |  |  |  |
|  | Never | | 1 (0.1) | 0 (0.0) | 1 (0.1) | .11^w^ |
|  | Mild to moderate | | 926 (99.6) | 161 (25.7) | 765 (30.2) |  |
|  | Heavy | | 3 (0.3) | 0 (0.0) | 3 (0.1) |  |
| Nutritional risk index, mean (SD) | | | 51.45 (6.69) | 51.53 (6.74) | 51.43 (6.67) | .77 |
| **Comorbidity** | | |  |  |  |  |
| Hypertension, n (%) | | | 1993 (63.1) | 420 (67.1) | 1573 (62.1) | .02 |
| Peripheral artery disease, n (%) | | | 64 (2.0) | 23 (3.7) | 41 (1.6) | .002 |
| COPD^f^, n (%) | | | 88 (2.8) | 20 (3.2) | 68 (2.7) | .58 |
| Dyslipidemia, n (%) | | | 1832 (58.0) | 356 (56.9) | 1476 (58.3) | .55 |
| Atrial fibrillation, n (%) | | | 129 (4.1) | 39 (6.2) | 90 (3.6) | .004 |
| Heart failure, n (%) | | | 131 (4.1) | 50 (8.0) | 81 (3.2) | <.001 |
| Chronic kidney disease, n (%) | | | 189 (6.0) | 63 (10.1) | 126 (5.0) | .001 |
| Dialysis, n (%) | | | 81 (2.6) | 32 (5.1) | 49 (1.9) | <.001 |
| Cerebrovascular accident, n (%) | | | 260 (8.2) | 63 (10.1) | 197 (7.8) | .08 |
| Diabetes mellitus, n (%) | | |  |  |  |  |
|  | | None | 2079 (65.8) | 361 (57.7) | 1718 (67.8) | <.001 |
|  | | Without medication | 137 (4.3) | 16 (2.6) | 121 (4.8) |  |
|  | | With OHA^g^ | 794 (25.1) | 207 (33.1) | 587 (23.2) |  |
|  | | With insulin | 149 (4.7) | 42 (6.7) | 107 (4.2) |  |
| **PCI-related variables** | | |  |  |  |  |
| ACS subtypes, n (%) | | |  |  |  |  |
|  | | Unstable angina | 1703 (53.9) | 363 (58.0) | 1340 (52.9) | .03 |
|  | | NSTEMI^h^ | 753 (23.8) | 125 (20.0) | 628 (24.8) |  |
|  | | STEMI^i^ | 703 (22.3) | 138 (22.0) | 565 (22.3) |  |
| Admission route, n (%) | | |  |  |  |  |
|  | | Outpatient department | 1557 (49.3) | 330 (52.7) | 1227 (48.4) | .15^w^ |
|  | | Emergency department | 1592 (50.4) | 293 (46.8) | 1299 (51.3) |  |
|  | | Non-acute transfer | 4 (0.1) | 2 (0.3) | 2 (0.1) |  |
|  | | Acute transfer | 4 (0.1) | 1 (0.2) | 3 (0.1) |  |
| Fluoro duration (min), mean (SD) | | | 31.73 (436.03) | 28.73 (25.27) | 32.49 (487.69) | .70 |
| Contrast volume (ml), mean (SD) | | | 264.93 (130.15) | 300.66 (140.38) | 256.10 (125.98) | <.001 |
| Catheterization status, n (%) | | |  |  |  |  |
|  | | Elective | 2067 (65.5) | 426 (68.1) | 1641 (64.8) | .06^w^ |
|  | | Urgent | 361 (11.4) | 51 (8.1) | 310 (12.2) |  |
|  | | Emergent | 726 (23.0) | 148 (23.6) | 578 (22.8) |  |
|  | | Salvage | 3 (0.1) | 1 (0.2) | 2 (0.2) |  |
| Access route, n (%) | | |  |  |  |  |
|  | | Radial artery | 346 (11.0) | 59 (9.4) | 287 (11.3) | .33^w^ |
|  | | Femoral artery | 2810 (89.0) | 566 (90.4) | 2244 (88.6) |  |
|  | | Other | 3 (0.1) | 1 (0.2) | 2 (0.1) |  |
| IABP^j^ used, n (%) | | | 109 (3.5) | 40 (6.4) | 69 (2.7) | <.001 |
| LVEF^k^ (%), mean (SD) | | | 60.39 (12.46) | 59.50 (12.88) | 60.64 (12.33) | .14 |
| RWMA^l^, n (%) | | | 532 (16.8) | 120 (19.2) | 412 (16.3) | .09 |
| ST segment in electrocardiogram, n (%) | | |  |  |  |  |
|  | | Normal | 1431 (45.3) | 271 (43.3) | 1160 (45.8) | .39 |
|  | | ST depression | 177 (5.6) | 43 (6.9) | 134 (5.3) |  |
|  | | ST elevation | 764 (24.2) | 153 (24.4) | 611 (24.1) |  |
|  | | Nonspecific | 787 (24.9) | 159 (25.4) | 628 (24.8) |  |
| Severity CAD, n (%) | | |  |  |  |  |
|  | | Insignificant | 15 (0.5) | 2 (0.3) | 13 (0.5) | <.001 |
|  | | 1 vessel disease | 837 (26.5) | 111 (17.7) | 726 (28.7) |  |
|  | | 2 vessel disease | 1026 (32.5) | 177 (28.3) | 849 (33.5) |  |
|  | | 3 vessel disease | 1281 (40.5) | 336 (53.7) | 945 (37.3) |  |
| TIMI^m^ flow grade, n (%) | | |  |  |  |  |
|  | | Grade 0 | 77 (2.5) | 18 (2.9) | 59 (2.3) | .90 |
|  | | Grade 1 | 18 (0.6) | 3 (0.5) | 15 (0.6) |  |
|  | | Grade 2 | 60 (1.9) | 13 (2.1) | 47 (1.9) |  |
|  | | Grade 3 | 2957 (95.0) | 584 (93.3) | 2373 (93.7) |  |
| Left main disease, n (%) | | | 232 (7.3) | 62 (9.9) | 170 (6.7) | .008 |
| PCI-Left main, n (%) | | | 98 (3.1) | 22 (3.5) | 76 (3.0) | .51 |
| PCI-LAD^o^, n (%) | | | 1648 (52.2) | 311 (49.7) | 1337 (52.8) | .16 |
| PCI-Left circumflex artery, n (%) | | | 569 (18.0) | 126 (20.1) | 443 (17.5) | .12 |
| PCI-Right coronary artery, n (%) | | | 861 (27.3) | 176 (28.1) | 685 (27.0) | .59 |
| **Laboratory test** | | |  |  |  |  |
| Total cholesterol (mg/dL), mean (SD) | | | 170.98 (44.98) | 170.23 (46.81) | 171.17 (44.52) | .66 |
| HDL-C^p^ (mg/dL), mean (SD) | | | 41.72 (10.99) | 40.49 (10.63) | 42.02 (11.07) | .03 |
| LDL-C^q^ (mg/dL), mean (SD) | | | 100.00 (37.93) | 103.27 (41.52) | 99.18 (36.95) | .12 |
| Triglyceride (mg/dL), mean (SD) | | | 131.35 (83.52) | 135.84 (99.20) | 130.23 (79.13) | .37 |
| CKMB^r^ (ng/ml), mean (SD) | | | 48.66 (117.78) | 47.96 (111.02) | 48.81 (119.25) | .90 |
| Troponin I (ng/ml), mean (SD) | | | 19.51 (242.89) | 13.97 (38.08) | 20.49 (263.08) | .41 |
| Creatinine (mg/dL), mean (SD) | | | 1.16 (1.14) | 1.41 (1.65) | 1.10 (0.96) | <.001 |
| GFR^s^ (ml/min/1.73㎡), mean (SD) | | | 75.93 (26.90) | 70.68 (28.73) | 77.25 (26.26) | <.001 |
| HbA1c (%), mean (SD) | | | 6.63 (1.41) | 6.86 (1.58) | 6.57 (1.36) | .02 |
| Uric acid (mg/dL), mean (SD) | | | 5.51 (1.89) | 5.73 (1.77) | 5.45 (1.91) | .001 |
| Hemoglobin (g/dL), mean (SD) | | | 13.31 (1.99) | 13.10 (2.01) | 13.36 (1.98) | .003 |
| CRP^t^ (mg/dL), mean (SD) | | | 1.30 (3.67) | 1.39 (3.68) | 1.28 (3.67) | .61 |
| Albumin (g/dL), mean (SD) | | | 3.81 (0.38) | 3.76 (0.37) | 3.83 (0.39) | <.001 |
| **Medication** | | |  |  |  |  |
| Antiplatelets, n (%) | | | 2932 (92.8) | 608 (97.1) | 2324 (91.7) | <.001 |
| GpIIb/IIIa inhibitors, n (%) | | | 208 (6.6) | 43 (6.9) | 165 (6.5) | .82 |
| Statins, n (%) | | | 1706 (54.0) | 337 (53.8) | 1369 (54.0) | .96 |
| ACEi^u^s, n (%) | | | 366 (11.6) | 94 (15.0) | 272 (10.7) | .003 |
| Angiotensin II receptor blockers, n (%) | | | 711 (22.5) | 160 (25.6) | 551 (21.8) | .047 |
| Beta blockers, n (%) | | | 1028 (32.5) | 221 (35.3) | 807 (31.9) | .11 |
| Calcium channel blockers, n (%) | | | 836 (26.5) | 179 (28.6) | 657 (25.9) | .19 |
| Nitrates, n (%) | | | 1992 (63.1) | 401 (64.1) | 1591 (62.8) | .59 |
| **Medication adherence** | | |  |  |  |  |
| MRCI^v^ on discharge, mean (SD) | | | 25.16 (11.52) | 26.14 (12.68) | 24.91 (11.20) | .03 |
| MRCI changed, mean (SD) | | | 3.64 (13.35) | 4.35 (13.93) | 3.46 (13.19) | .16 |

^a^PCI: percutaneous coronary intervention

^b^ACS: acute coronary syndrome

^c^MACE: major adverse cardiovascular event

^d^SD: standard deviation

^e^CAD: coronary artery disease

^f^COPD: chronic obstructive pulmonary disease

^g^OHA: oral hypoglycemic agents

^h^NSTEMI: non-ST-elevation myocardial infarction

^i^STEMI: ST-elevation myocardial infarction

^j^IABP: intra-aortic balloon pump

^k^LVEF: left ventricular ejection fraction

^l^RWMA: regional wall motion abnormality

^m^TIMI: thrombolysis in myocardial infarction

^o^LAD: left anterior descending coronary artery

^p^HDL-C: high density lipoprotein cholesterol

^q^LDL-C: low density lipoprotein cholesterol

^r^CKMB: creatine kinase myocardial bound

^s^GFR: glomerular filtration rate

^t^CRP: C-reactive protein

^u^ACEi: angiotensin converting enzyme inhibitor

^v^MRCI: medication regimen complexity index

^w^Fisher’s exact test
