# Supplementary material for: Risk Prediction of Major Adverse Cardiovascular Events Within One Year After Percutaneous Coronary Intervention in Patients With Acute Coronary Syndrome: Machine Learning–Based Time-to-Event Analysis
Source: JMIR Med Inform. 2025 Nov 27;13:e81778. doi: 10.2196/81778 (PMC12699253; doi:10.2196/81778)
Supplement: Multimedia Appendix 5 [file medinform_v13i1e81778_app5.docx]

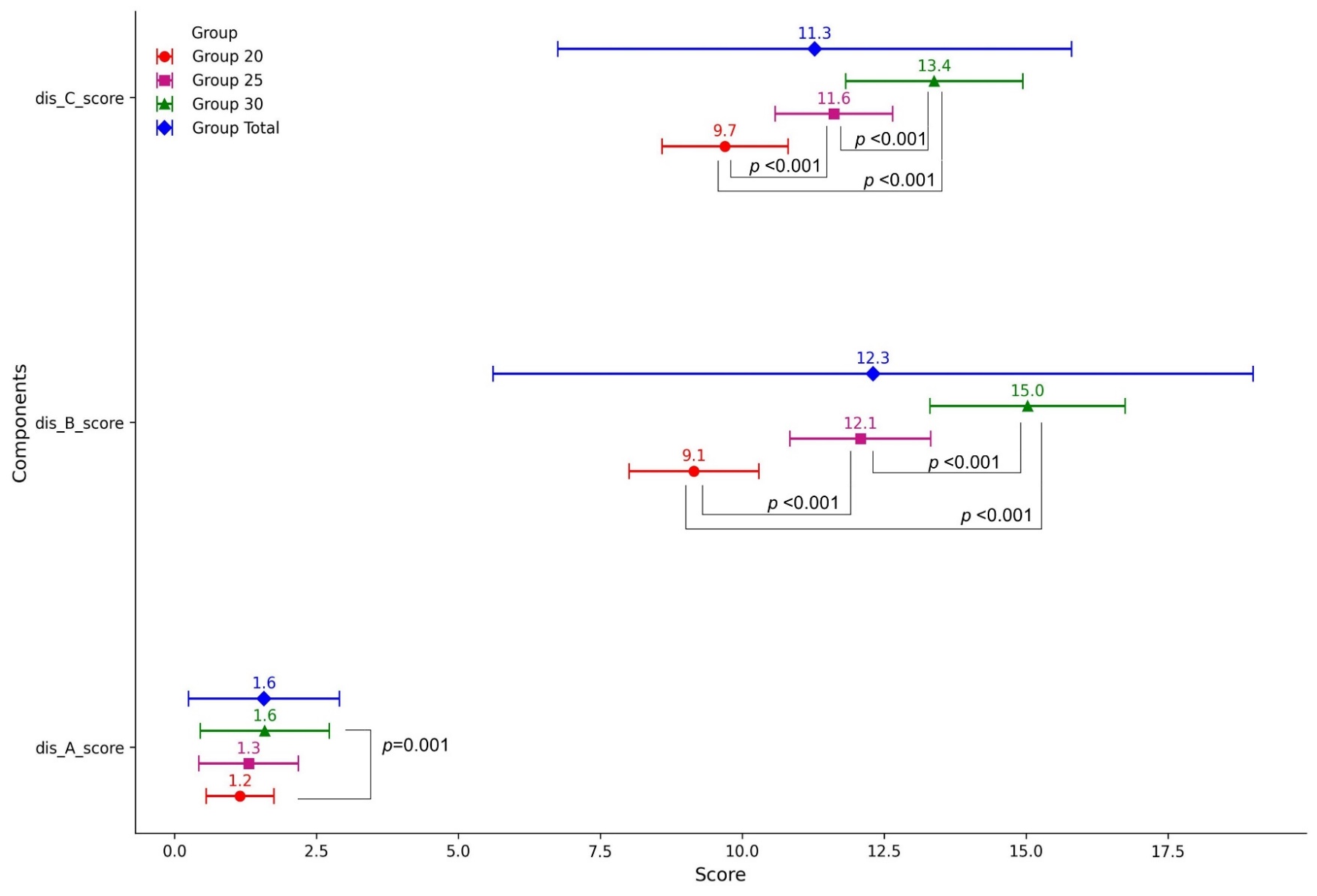


Multimedia Appendix 5. Comparison of three MRCI components across score-based subgroups (20, 25, and 30 points) and the total cohort.

*P* value represent post hoc comparisons using the Scheffé methods following ANOVA.
